# TSF-MDD: A Deep Learning Approach for Electroencephalography-Based Diagnosis of Major Depressive Disorder with Temporal–Spatial–Frequency Feature Fusion

**DOI:** 10.3390/bioengineering12020095

**Published:** 2025-01-21

**Authors:** Wei Gan, Ruochen Zhao, Yujie Ma, Xiaolin Ning

**Affiliations:** 1 School of Instrumentation Science and Optoelectronic Engineering, Beihang University, Beijing 100191, China; ganwei_edu@buaa.edu.cn (W.G.); zhaoruochen@buaa.edu.cn (R.Z.); mayujie@buaa.edu.cn (Y.M.); 2 Hangzhou Institute of National Extremely-Weak Magnetic Field Infrastructure, Hangzhou 310000, China; 3 Hefei National Laboratory, Gaoxin District, Hefei 230088, China

**Keywords:** major depression disorder (MDD), electroencephalography (EEG), deep learning, temporal–spatial–frequency

## Abstract

Major depressive disorder (MDD) is a prevalent mental illness characterized by persistent sadness, loss of interest in activities, and significant functional impairment. It poses severe risks to individuals’ physical and psychological well-being. The development of automated diagnostic systems for MDD is essential to improve diagnostic accuracy and efficiency. Electroencephalography (EEG) has been extensively utilized in MDD diagnostic research. However, studies employing deep learning methods still face several challenges, such as difficulty in extracting effective information from EEG signals and risks of data leakage due to experimental designs. These issues result in limited generalization capabilities when models are tested on unseen individuals, thereby restricting their practical application. In this study, we propose a novel deep learning approach, termed TSF-MDD, which integrates temporal, spatial, and frequency-domain information. TSF-MDD first applies a data reconstruction scheme to obtain a four-dimensional temporal–spatial–frequency representation of EEG signals. These data are then processed by a model based on 3D-CNN and CapsNet, enabling comprehensive feature extraction across domains. Finally, a subject-independent data partitioning strategy is employed during training and testing to eliminate data leakage. The proposed approach achieves an accuracy of 92.1%, precision of 90.0%, recall of 94.9%, and F1-score of 92.4%, respectively, on the Mumtaz2016 public dataset. The results demonstrate that TSF-MDD exhibits excellent generalization performance.

## 1. Introduction

Major depressive disorder (MDD) is a significant public health issue affecting modern society. MDD is characterized by at least one discrete depressive episode lasting at least 2 weeks and involving clear-cut changes in mood, interests and pleasure, changes in cognition, and vegetative symptoms [1]. Currently, the diagnosis of MDD primarily relies on physicians using medical rating scales in combination with their professional experience to perform subjective and comprehensive evaluations of patients. However, there is still a lack of mature, objective diagnostic methods for practical application. Various neuroimaging techniques are available to explore the neural mechanisms, cognitive functions, and diagnostic approaches of psychiatric disorders. Among these, electroencephalography (EEG) [2] stands out due to its millisecond-level temporal resolution, enabling non-invasive functional assessments of the brain. EEG is portable, cost-effective, and can be administered in a variety of settings, including primary care clinics or remote areas with limited access to specialized mental healthcare. Research indicates that most psychological and cognitive activities are reflected in neuronal electrophysiological signals, and EEG can capture these signals [3], making it a valuable tool for MDD diagnostic research. The accessibility and affordability of EEG could revolutionize the diagnostic process, particularly in resource-limited environments, by providing a faster and more objective means of identifying MDD.

Studies have found differences between MDD patients and healthy controls in terms of energy distribution across different frequency bands, brain functional connectivity, and hemispheric asymmetry [4]. These differences in EEG signals between MDD patients and healthy individuals enable the diagnosis of MDD through EEG signal classification and recognition. Traditional analysis methods rely heavily on manual interpretation of EEG signals, which is time-consuming and requires a high level of expertise. With advancements in artificial intelligence (AI), machine learning-based methods have emerged as a promising approach to improve efficiency while maintaining accuracy. Machine learning has increasingly been applied in the diagnosis and treatment of psychiatric disorders. For example, Reference [5] employs machine learning methods to diagnose MDD, while Reference [6] uses machine learning to explore how to predict outcomes for treatment-resistant depression. Current machine learning approaches include traditional machine learning and deep learning [7,8]. Traditional machine learning involves manual feature engineering, where features are extracted from data and fed into classifiers for results. However, these methods heavily depend on manually selected features, which may require adjustments for different psychiatric disorders. Deep learning, by contrast, automates feature engineering, enabling the model to process raw data with minimal pre-processing to solve complex classification tasks. Although deep learning has found broad applications in the medical field, its application in mental disorder diagnosis is still in its early stages. Several challenges remain before deep learning can be effectively translated into clinical practice.

Firstly, extracting effective information remains a significant challenge. The complexity of diagnosing MDD is further compounded by temperamental and neurophysiological variabilities, as discussed by Favaretto et al. [9], which underscores the need for comprehensive diagnostic approaches that can address the multifaceted nature of mental disorders. EEG signals are typically multichannel, nonlinear temporal signals, where each channel corresponds to a distinct spatial location, embedding spatial features of brain activity. Furthermore, EEG data exhibit temporal, spatial, and frequency-domain patterns associated with specific cognitive processes [10]. Many current studies focus exclusively on temporal, frequency, or spatial features. Some studies attempt to explore the relationships between channels using functional connectivity matrices [11,12], but they may overlook valuable time-frequency features inherent in the raw signals. Therefore, it is necessary to fully exploit the multichannel EEG signals by extracting and integrating features across temporal, spatial, and frequency domains.

Secondly, data leakage in experimental methods poses a problem. EEG signals exhibit significant individual differences, influenced by factors such as gender, age, mental health status, and brain anatomy [13]. Many studies [14,15,16,17,18,19,20,21] adopt subject-dependent training and testing set partitioning, where EEG data segments from the same individual may appear in both the training and testing sets. This approach causes models to learn individual-specific features rather than generalized features independent of the subject. However, in real-world applications, models need to generalize to unseen individuals. To address this, a subject-independent partitioning method should be adopted, ensuring that EEG data from the same subject appear only in either the training or the testing set. This strategy eliminates individual differences that could bias classification results. Table 1 summarizes recent representative studies comparing subject-dependent and subject-independent experimental results. Subject-dependent studies generally report higher accuracy, with some achieving up to 99.90% [18]. In contrast, subject-independent studies report relatively lower accuracy, with the lowest being 80.74% [22].

The issues mentioned above result in poor generalization performance of the model when applied to new subjects. Therefore, it is crucial to explore how to effectively extract information from EEG signals to improve the model’s generalization performance under subject-independent conditions, thereby promoting practical applications. In this context, the contribution of this study lies in providing an innovative method that integrates temporal, spatial, and frequency-domain features from multichannel EEG signals, aimed at improving the diagnosis of MDD. We first designed a data reconstruction scheme that maps multichannel EEG data onto a 2D electrode layout, and then extracts frequency-specific signals through band-pass filtering, ultimately forming 4D data to represent multichannel temporal signals across time, space, and frequency domains. Based on the input data structure, we proposed a classification model based on 3D-CNN and Capsule Networks (CapsNet). The 3D-CNN was introduced to adapt to the 4D data format and extract features from different receptive fields, while the CapsNet was further employed to capture higher-order spatial features. We conducted experiments using the publicly available Mumtaz2016 dataset with a subject-independent partitioning method, demonstrating the excellent performance of the proposed model. We expect that the proposed method can provide a more robust and objective approach for MDD diagnosis, which is particularly important in environments with limited access to mental health professionals. By integrating this method into existing clinical pathways, it can assist clinicians in making more accurate diagnoses, reducing the risk of misdiagnosis and underdiagnosis.

## 2. Related Works

Research in cognitive neuroscience indicates that individuals with major depressive disorder (MDD) exhibit structural and functional abnormalities in the brain compared to healthy individuals [25]. Many studies have sought to identify effective biomarkers for MDD by analyzing differences in resting-state or task-state EEG signals using statistical methods. Machine learning approaches have also been widely applied in EEG analysis. Commonly used classification algorithms include support vector machines (SVMs), k-nearest neighbors (KNNs), linear discriminant analysis (LDA), and decision trees (DTs). Among these, SVM is the most frequently adopted algorithm in EEG-based MDD diagnosis, consistently demonstrating excellent performance across studies [5,26,27,28]. Traditional machine learning approaches rely on manually selecting features, typically extracted from EEG data across spectral, statistical, nonlinear, and time-frequency domains. Common features include power spectral density, mean values, fractal dimension, hemispheric asymmetry, and functional connectivity matrices of brain networks. However, EEG data are high-dimensional, with multiple channels capturing distinct spatial and temporal brain activity, leading to an overwhelming number of features. EEG signals are also dynamic and nonlinear, exhibiting time-varying patterns that are difficult to model with linear approaches. This complexity, along with substantial inter-individual variability in brain structure, cognitive states, and psychiatric conditions, makes it challenging to identify features that generalize across subjects. Additionally, the theoretical foundations of psychiatric disorders remain insufficient to guide feature selection, complicating the development of reliable and universally applicable biomarkers. However, deep learning, with its capability for automated feature extraction, offers a solution to this limitation.

EEG information is generally expressed across the temporal, spatial, and frequency domains. Consequently, some researchers have focused on extracting features from specific domains. Some studies treat multichannel EEG signals as 2D images and input them into convolutional neural networks (CNNs) primarily focusing on temporal features. For instance, Seal et al. [15] proposed the DeprNet, achieving 99.37% accuracy under subject-dependent conditions and 91.4% accuracy under subject-independent conditions on their private dataset. The spatial characteristics of EEG signals are represented by the spatial layout of electrodes and are often analyzed through functional connectivity networks. Li et al. [22] used functional connectivity matrices across five EEG frequency bands as input to CNNs, distinguishing between patients with MDD and healthy individuals. Dang et al. [16] combined frequency-band-specific brain networks with CNNs to detect MDD, achieving 97.27% accuracy under subject-dependent conditions on the public Mumtaz2016 dataset [29]. Chen et al. [24] introduced the SGP-SL model, employing self-attention mechanisms to construct adjacency matrices and analyze channel relationships. This approach achieved superior performance on classification and regression tasks using the public MODMA dataset [30]. Similarly, Xia et al. [23] utilized a self-attention mechanism to automatically learn the latent relationships among multichannel EEG signals.

Although extracting features from brain network connectivity matrices highlights spatial information, it overlooks other valuable information in the raw signals. Some researchers have combined temporal and spatial domains in their studies. For example, Saeedi et al. [17] combined LSTM with 1D-CNNs and 2D-CNNs to extract effective temporal and spatial features for MDD detection based on effective EEG connectivity. However, they did not consider frequency-domain information. Yan et al. [31] developed a multi-scale convolutional recurrent neural network (MCRNN), which integrated CNNs and GRUs to enhance sensitivity to long-term memory. This approach utilized spatiotemporal information from resting-state EEG to classify schizophrenia, bipolar disorder, MDD, and healthy controls, achieving an accuracy of 68.2%, but neglected frequency-domain features. Sharma et al. [18] applied Short-Time Fourier Transform (STFT) to obtain spectrograms from raw EEG data, which served as input to a model combining CNN and LSTM. This method emphasized frequency-domain features but overlooked other domains.

Emotional dysfunction is a common symptom of mental disorders, and research in EEG-based emotion recognition offers insights for addressing challenges in mental health diagnostics. Some studies have used 3D-CNNs to capture both temporal and spatial information. For example, Salama et al. [32] developed a 3D data representation for EEG input, allowing their proposed 3D-CNN model to extract temporal and spatial features. Liu et al. [33] designed the 3DCANN model to capture dynamic temporal relationships between EEG channels and spatial relationships within consecutive time segments of multichannel EEG signals. Capsule networks (CapsNet) have also been explored for emotion recognition, demonstrating superior performance in spatial feature extraction [34]. Liu et al. [35] applied a multi-level feature-guided CapsNet for multichannel EEG emotion recognition. Multi-level features were derived by combining shallow and deep features extracted by CNNs and inputting them into primary capsules. Guo et al. [36] used a CapsNet to classify emotions based on Granger causality matrices derived from wavelet-transformed EEG signals. However, this approach entirely excluded electrode spatial arrangement information, failing to fully leverage CapsNet’s advantages. Recently, Wang et al. [37] combined spatial features extracted by CapsNet with manually derived temporal features from EEG microstate analysis to classify generalized anxiety disorder. Although this marked the first application of CapsNet in mental health diagnostics, its reliance on manually extracted temporal features prevents end-to-end implementation. To address the limitations of existing approaches and capture complementary features across temporal, spatial, and frequency domains in multichannel EEG signals, this study introduces an MDD diagnostic approach that integrates feature extraction from all three domains.

## 3. Materials and Methods

### 3.1. Design of Data Structure

**Figure 1:**
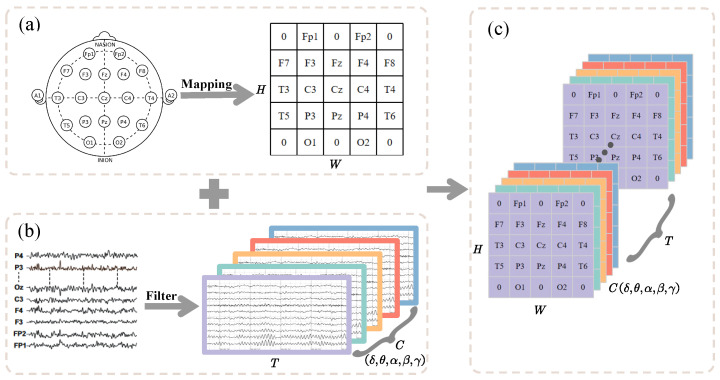
Schematic diagram of data structure reconstruction. (**a**) Spatial mapping; (**b**) frequency domain filtering; (**c**) 4D time–spatial–frequency data representation.

To fully extract information from EEG Signals, we designed a data structure reconstruction scheme that explicitly represents the temporal, spatial, and frequency information of multichannel EEG signals. For each subject, the EEG data structure at time point *t* after denoising can be simply expressed as $V_{t}=\left\{s_{1}^{i},s_{2}^{i},\ldots ,s_{t}^{i}\right\}$, where $s_{t}^{i}$ represents the *i*-th channel at time point *t*. In general, studies input the EEG signal directly into deep learning networks for feature extraction. However, multichannel EEG signals follow a specific spatial arrangement. The experimental dataset adopts the 21-channel layout based on the international 10–20 system, where each channel is surrounded by neighboring channels at varying distances and orientations. The original EEG data structure fails to capture this spatial positioning. Therefore, in this study, the multichannel EEG time series is transformed into a 2D frame sequence. As shown in Figure 1a, after excluding the reference electrodes, the 19 active channels are mapped, and the projected matrix frame has a size of H×W (H,W represent the height and width of the projection matrix plane, respectively). The sequence of mapped data frames fills the unused electrode positions with zeros.

This transformation achieves the extraction of temporal and spatial features, but the information in the frequency domain remains absent. EEG signals are generally analyzed across different frequency bands. Studies have found that positive emotions in patients with MDD are positively correlated with the amplitude of the alpha band in the left frontal EEG [38]. Jaworska et al. [39] discovered that the theta power in resting-state EEG is higher in patients with depression than in healthy individuals. Siegle et al. [40], through an emotional word valence recognition task, found that MDD patients exhibit sustained and increased gamma power when processing negative words. In summary, studying MDD-related EEG features across basic frequency bands is effective [41]. Thus, the original EEG signals are passed through FIR band-pass filters with a Hanning window to extract time-series signals in five frequency bands, as shown in Figure 1b. Each frequency band signal $X_{j}$ is defined as follows:

(1) $$X_{j}=\left\{a_{jt}^{1},a_{jt}^{2},...,a_{jt}^{n-1},a_{jt}^{n}\right\},\;\left(j\in B\right)$$

where $B=\left\{\delta ,\;\theta ,\;\alpha ,\;\beta ,\;\gamma \right\}$, $a_{it}^{j}$ represents the sampling data of the *i*-th electrode channel in frequency band *j* at time point *t* after filtering.

By combining the spatial mapping and frequency filtering, we achieved the data structure shown in Figure 1c. Each data frame is represented as three-dimensional data of size H×W×C, and with a total of frames, the data form a four-dimensional structure.

### 3.2. Structure of the Model

After the aforementioned data structure reconstruction, for each subject $x_{\mathrm{sub}}\in R^{H\times W\times C\times T\times N}$, where *H* and *W* denote the height and width of the projection matrix plane. In this model, H=W=5. *C* represents the number of frequency bands, and in this model C=5. And *T* and *N* denote the length and number of consecutive EEG segments after slicing for each subject, respectively. In the experiment, *T* is set to 1280, while *N* varies depending on the subject.

The MDD automatic diagnosis problem under study can be regarded as a binary classification problem, where each subject corresponds to a label ${\hat{y}}_{\mathrm{sub}}\in \left\{0,1\right\}$, where 0 represents healthy controls (HCs), and 1 represents MDD patients. Each input sample $x\in R^{H\times W\times C\times T}$ corresponds to a label $\hat{y}={\hat{y}}_{\mathrm{sub}}$. The model functions as a classifier, mapping each input sample to its respective label: f:y=f(x),y∈[0,1].

**Figure 2:**
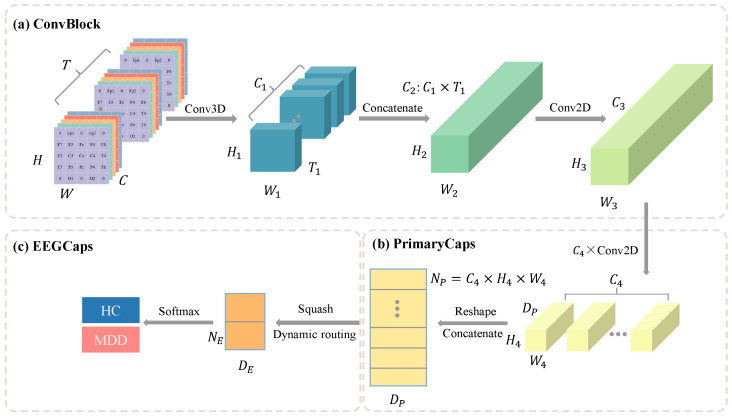
Overall structure of the model. (**a**) ConvBlock; (**b**) PrimaryCaps; (**c**) EEGCaps representation.

Figure 2 shows the overall structure of the model. The proposed model mainly consists of two modules: ConvBlock and CapsNet. The CapsNet module is further divided into two submodules: PrimaryCaps and EEGCaps. Table 2 outlines the specific layers and parameter configurations within the model.

#### 3.2.1. ConvBlock

ConvBlock includes a 3D convolutional layer (3D-CNN) and a subsequent 2D convolutional layer (2D-CNN), which bridges to the next module. The convolutional layers are followed by a Batch Normalization (BN) layer and a ReLU activation function. The BN layer aims to accelerate model convergence and avoid overfitting. The 3D-CNN serves as the interface module after data input. Its main purpose is to preserve temporal and spatial features of the input four-dimensional data and prepare them for the capsule layers. Standard convolutions are typically 2D-CNNs, which only focus on spatial information from single 2D images, suitable for visual tasks. However, in this study, the input data contain an additional time dimension, which can be interpreted as video information, making 3D-CNN more appropriate. The main advantage of 3D-CNN is its ability to capture temporal sequences, which is crucial for EEG signals as time-series data. For the input $x\in R^{H\times W\times C\times T}$, the 3D convolution kernel size can be defined as $K_{H}\times K_{W}\times K_{T}\times C$, where $K_{H},\;K_{W},\;K_{T}$ represent the kernel’s dimensions in spatial (height, width) and temporal directions, respectively. The 3D-CNN operation can be expressed as follows:

(2) $$y\left(i,j,k,l\right)=\sum_{m=0}^{K_{W}-1}\sum_{n=0}^{K_{H}-1}\sum_{o=0}^{K_{T}-1}\sum_{c=0}^{C-1}x\left(i+m,j+n,k+o,c\right)\cdot K\left(m,n,o,c\right)$$

where *y* represents the output feature map, (i,j,k) denotes the position in the output feature map, *l* indicates the output channel, (m,n,o) corresponds to the positions traversed by the convolution kernel in the spatial and temporal dimensions, and *c* represents the input channel being traversed.

#### 3.2.2. CapsNet

To further extract features, CapsNet is applied after ConvBlock. Compared to CNNs, CapsNet excels at spatial feature extraction, making it suitable for the data structure in this study. CapsNet comprises two components: PrimaryCaps and EEGCaps. PrimaryCaps is a convolutional capsule layer, which sequentially applies parallel 2D convolutions (with $C_{4}$ filters, each of size 3×3, and a stride of 1), reshapes the data, and performs concatenation operations to obtain $N_{P}\left(N_{P}=C_{4}\times H_{4}\times W_{4}\right)$ primary capsule vectors, each with a dimensionality of $D_{P}$. The length and orientation of each capsule vector represent the model’s representative features, providing richer information compared to the scalar output of traditional networks. EEGCaps integrates the capsule vector information from PrimaryCaps to form higher-level capsule vectors. Since this study focuses on a binary classification task, EEGCaps contains $N_{E}\left(N_{E}=2\right)$ capsule vectors, each with a dimensionality of $D_{E}$.

**Figure 3:**
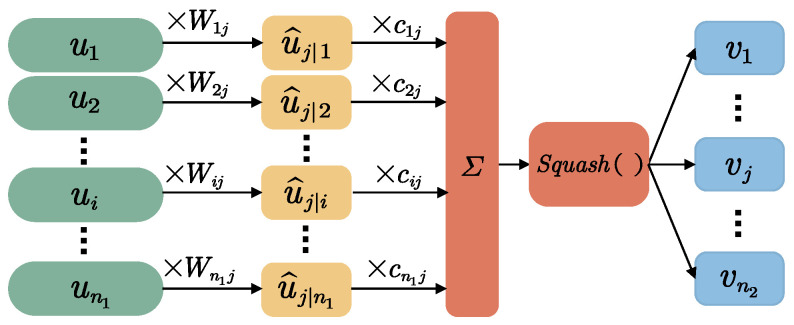
Dynamic routing algorithm.

The dynamic routing algorithm [42], illustrated in Figure 3, is used to integrate and filter features between PrimarvCaps and EEGCaps. For the *i*-th primary capsule, the affine transformation of its features $u_{i}\;\left(i=1,2,\ldots ,n_{1}\right)$ with a weight matrix $W_{ij}\;\left(j=1,\;2,\;\ldots ,\;n_{2}\right)$ produces a prediction vector ${\hat{u}}_{j|i}$:

(3) $${\hat{u}}_{j|i}=W_{ij}u_{i}$$

The input $s_{j}$ to EEGCaps is the weighted sum of ${\hat{u}}_{j|i}$:

(4) $$s_{j}=\sum_{i}c_{ij}{\hat{u}}_{j|i}$$

(5) $$c_{ij}=\frac{\exp\left(b_{ij}\right)}{\sum_{n}\exp\left(b_{in}\right)}$$

where $c_{ij}$ is the coupling coefficient determined by the iterative dynamic routing algorithm. $b_{ij}$ is the logarithmic prior probability of the combination of the *i*-th primary capsule and the *j*-th advanced capsule.

Next, the Squash function is used to limit the length of the advanced capsule output vector to between 0 and 1. The formula is as follows:

(6) $$v_{j}=\frac{∥s_{j}{∥}^{2}}{1+∥s_{j}{∥}^{2}}\frac{s_{j}}{∥s_{j}∥}$$

By calculating the current output $v_{j}$ and ${\hat{u}}_{j|i}$ to update $b_{i}j$ to iteratively update the coupling coefficient, the formula is as follows:

(7) $$b_{ij}\leftarrow b_{ij}+v_{j}{\hat{u}}_{j|i}$$

#### 3.2.3. Model Loss Function

Since CapsNet outputs a vector for each class, traditional loss functions have limitations. Therefore, the loss function used in this study is the Margin Loss [42], which is based on the idea of defining a margin boundary for each class. The model is expected to output class vectors that are close to this boundary while staying far from the boundaries of other classes. The margin loss function for each class *l* can be expressed as follows:

(8) $$L_{l}=T_{l}\cdot \max(0,m^{+}-\left|\right|v_{l}{\left||\right)}^{2}+\lambda \left(1-T_{l}\right)\cdot \max(0,||v_{l}\left|\right|-m^{-}{)}^{2}$$

where $v_{l}$ represents the capsule vector for class *l*, and $T_{l}$ is the indicator function for class *l* (equal to 1 if the class is present, and 0 otherwise). For a given class, if the true label is positive (correct classification), the magnitude of the output vector $\left|\right|v_{l}\left|\right|$ should be as large as possible, approaching the upper margin $m^{+}$. If the true label is negative (incorrect classification), the magnitude of the output vector should be as small as possible, moving away from the lower margin $m^{-}$. The values of $m^{+}$ and $m^{-}$ are typically set to 0.9 and 0.1, respectively. λ is a weight parameter used to balance the loss for positive and negative samples, typically set to 0.5.

The total loss function is the sum of the losses for all output capsule vectors:

(9) $$L_{\mathrm{total}}=\sum_{l=1}^{n}L_{l}$$

where *n* is the number of output capsule vectors.

## 4. Experiment Results and Discussion

### 4.1. Dataset and Pre-Processing

#### 4.1.1. Dataset

The dataset used in the experiments is Mumtaz2016 [29], which includes EEG data from 34 MDD patients and 30 healthy controls (HCs) across 19 channels. The dataset is open access and freely available for research purposes. The participants were recruited from outpatient clinics at Universiti Sains Malaysia Hospital (HUSM), with MDD diagnoses based on DSM-IV criteria. Among the MDD patients, there were 17 males and 17 females, with an average age of 40.3±12.9 years; the HC group comprised 21 males and 9 females, with an average age of 38.3±15.6 years. After excluding subjects with incomplete EEG data, the dataset contained 5 min of eyes-closed resting-state EEG recordings from 30 MDD patients and 28 HC participants for the experiments.

#### 4.1.2. Pre-Processing

Following the exclusion of subjects with incomplete EEG data, 30 MDD patients and 28 HC participants’ 5-minute eyes-closed resting-state EEG data were used in the experiments. The EEG recordings were acquired using 19 electrodes (Fp1, F3, C3, P3, O1, F7, T3, T5, Fz, Fp2, F4, C4, P4, O2, F8, T4, T6, Cz, Pz) and reference electrodes at both earlobes (A1, A2), with a sampling frequency of 256 Hz. The data analysis was performed with the MNE-Python 1.9.0 [43]. The preprocessing steps were as follows:

1. Band-pass and Notch Filtering: A 0.5–70 Hz band-pass filter was applied to remove high-frequency noise and low-frequency drift. A 50 Hz notch filter was used to eliminate power line interference [5];
2. Artifact Removal: Independent Component Analysis (ICA) [44] was applied to remove artifacts such as ocular, muscular, and cardiac signals;
3. Data Segmentation: Continuous EEG data were segmented into non-overlapping 5-second windows, resulting in samples with 1280 data points (5s×256Hz);
4. Noise Removal: Segments with amplitudes exceeding ±100 μV were considered noisy and discarded;
5. Re-referencing: The signal average across all electrodes was used as the reference signal;
6. Data Standardization: Z-score normalization was applied to transform signal values to have a mean of 0 and a standard deviation of 1, ensuring consistent data magnitudes for model training;
7. Data Structure Reconstruction: The reconstructed data structure followed the method described in Section 3.2, with five frequency bands defined as follows: δ(0.1-4Hz), θ(4-8Hz),α(8-13Hz), β(13-30Hz), γ(30-100Hz). The final dataset contained 3237 samples, with 1640 from HC and 1597 from MDD participants.

#### 4.1.3. Partitioning Method

To prevent data leakage and simulate real-world application scenarios, subject-independent 5-fold cross-validation was employed. This ensured that samples from the same subject appeared exclusively in either the training or validation set.

### 4.2. Experiment Setup

The experiments were implemented using the PyTorch library. The model was optimized with the Adam optimizer, using a learning rate of 1 × 10^−4^. An early stopping strategy was adopted to prevent overfitting. The batch size for model training was set to 32. Common binary classification metrics were used to evaluate performance, including accuracy (Acc), precision (Pre), recall (Rec) and F1-score (F1).

### 4.3. Senstivity Analysis of Hyperparameters

The proposed model involves several custom hyperparameters that cannot be optimized directly by the model itself and require manual tuning. This section investigates the impact of key hyperparameters on the model’s performance.

#### 4.3.1. Number of Filters in 3D-CNN

**Figure 4:**
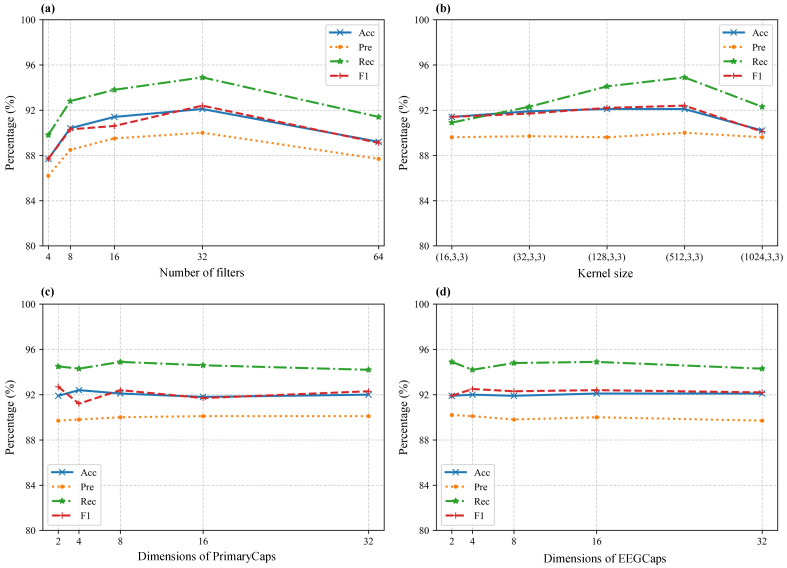
Hyperparameter sensitivity analysis result. (**a**) Number of filters in 3D-CNN; (**b**) Kernel size of 3D-CNN; (**c**) Capsule vector dimensions in PrimaryCaps; (**d**) Capsule vector dimensions in EEGCaps.

As the front-end layer of the model, the number of filters in the 3D-CNN significantly affects feature extraction. As shown in Figure 4a, the filter numbers were tested across typical values: 4, 8, 16, 32, and 64, while keeping all other hyperparameters consistent with Table 2. The results indicate that the model’s performance improves initially as the number of filters increases but decreases afterward. This trend suggests that an insufficient number of filters hinders the extraction of sufficient features, whereas an excessive number of filters leads to overfitting on the training set, reducing test performance. The optimal filter count for the model is set at 32.

#### 4.3.2. Kernel Size of 3D-CNN

The kernel size of the 3D-CNN, particularly along the temporal dimension, has a notable influence on model performance. To explore this effect, experiments were conducted by varying only the kernel size along the temporal dimension, while maintaining other hyperparameters as listed in Table 2. The kernel sizes tested were (16, 3, 3), (32, 3, 3), (128, 3, 3), (512, 3, 3), and (1024, 3, 3), as shown in Figure 4b. Results demonstrate that this parameter moderately affects performance. Increasing the temporal kernel size from (16, 3, 3) to (512, 3, 3) enhances performance by less than 1%, whereas increasing it further to (1024, 3, 3) causes performance to drop by over 2%. This phenomenon can be explained by convolutional principles: larger kernels expand the receptive field but reduce the network’s sensitivity to fine-grained features. Consequently, selecting an appropriate kernel size is crucial. The model uses (512, 3, 3) as the optimal kernel size.

#### 4.3.3. Dimensions of Capsule Vectors in CapsNet

The key hyperparameter in the CapsNet module is the dimensions of the capsule vectors. Experiments were conducted by varying the dimensions of the capsule vectors in both the PrimaryCaps and EEGCaps layers, while keeping all other hyperparameters consistent with Table 2. The results, as illustrated in Figure 4c,d, reveal that the dimensions of the capsule vectors have minimal impact on the model’s performance. Thus, a reasonable choice for this parameter suffices. The model sets the dimensions of capsule vectors to 8 for the PrimaryCaps layer and 16 for the EEGCaps layer.

### 4.4. Ablation Study

**Figure 5:**
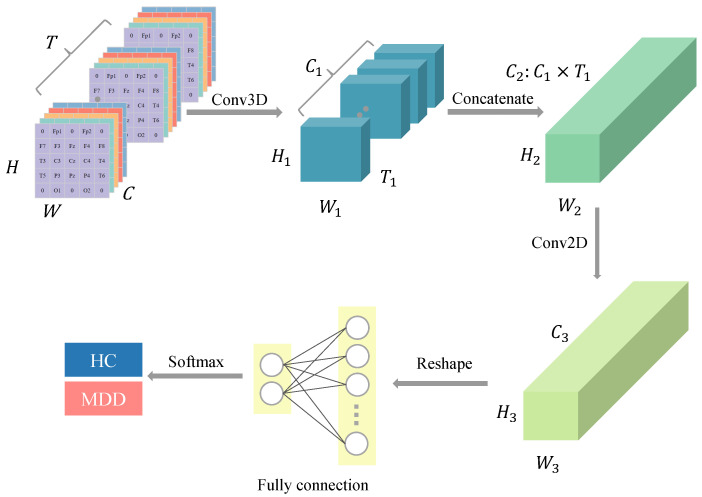
Structure of Conv-FC.

To investigate the contribution of each module to the overall model, ablation experiments were conducted. Since the model primarily consists of the ConvBlock and CapsNet modules, comparisons were made between a model containing only the ConvBlock module and the proposed model, which integrates CapsNet after the ConvBlock. Notably, to enable classification directly after the ConvBlock module, a fully connected layer and a softmax function were appended to the ConvBlock, forming the Conv-FC model, as illustrated in Figure 5. As shown in Table 3, the ablation results reveal that, compared to the proposed model, Conv-FC exhibits approximately a 1% decrease in both F1 and Acc, and a more than 2% drop in Rec. This indicates that the inclusion of CapsNet enables the extraction of more features beneficial for diagnosing MDD, thereby enhancing the overall model performance.

### 4.5. Comparative Experiments

To verify the superior performance of the proposed TSF-MDD model, we conducted comparative experiments from two perspectives: comparing different input data formats and benchmarking against representative methods in the field.

#### 4.5.1. Comparison of Different Data Types

The first set of experiments compared the impact of different input data types on model performance, with the results summarized in Table 4. We selected EEGNet [45,46] as the baseline model, which is based on a CNN architecture and has demonstrated strong performance across various EEG-related tasks. EEGNet uses raw multichannel EEG signals as input, focusing on extracting temporal features. The TS-MDD variant represents a model with the same architecture as the proposed TSF-MDD model but with input data limited to temporal and spatial dimensions, without multi-band frequency combinations (i.e., the channel dimension is set to 1). As shown in Table 4, the proposed data input scheme significantly outperforms both the baseline EEGNet model and TS-MDD. This highlights the effectiveness of combining temporal, spatial, and frequency features in improving model performance.

#### 4.5.2. Comparison with Representative Methods

In the second set of experiments, the proposed model was compared with representative methods from the literature. Many prior studies used subject-dependent conditions or private datasets, making direct comparison with this study infeasible. Thus, we reproduced these models’ experiments on the public Mumtaz2016 dataset for a fair comparison. Table 5 summarizes the performance comparison between the proposed TSF-MDD model and the competing methods. The results indicate that the proposed model achieves the best performance in terms of Acc, Rec, and F1. The higher Acc indicates a lower misdiagnosis rate. The higher Rec rate reflects fewer missed diagnoses, critical for disease screening tasks.

Among the traditional machine learning methods, SVM shows the lowest performance, with gaps exceeding 10% in Acc, Rec, and F1 compared to the proposed model. This may stem from inaccuracies in manually selected features, leading to the loss of important information. Other deep learning methods implemented different architectures. EEGNet and DeprNet, both based on 1D-CNN, show slightly better performance for DeprNet than EEGNet, but still lag over 4% behind the proposed model. These methods primarily rely on temporal-domain features and fail to fully leverage spatial and frequency-domain information. 1D-CNN-LSTM, which integrates LSTM for extracting long-range temporal features, performs similarly to EEGNet and DeprNet but is still 3% behind the proposed model. This suggests that relying solely on temporal features may lead to overfitting and limited performance improvement. InceptionNet achieves comparable performance to the 1D-CNN-based models. Self-Attention-CNN achieves the best performance among the comparison models due to its attention mechanism, which captures inter-channel relationships and reflects functional brain connectivity. However, it still lags behind the proposed model by approximately 2% across key metrics. The experimental results underscore the strength of the proposed TSF-MDD model.

In summary, by designing a novel input data structure to extract features from temporal, spatial, and frequency domains, and by incorporating CapsNet for superior spatial feature extraction compared to CNNs, the proposed model achieves enhanced generalization and performance.

### 4.6. Feature Visualization Analysis

**Figure 6:**
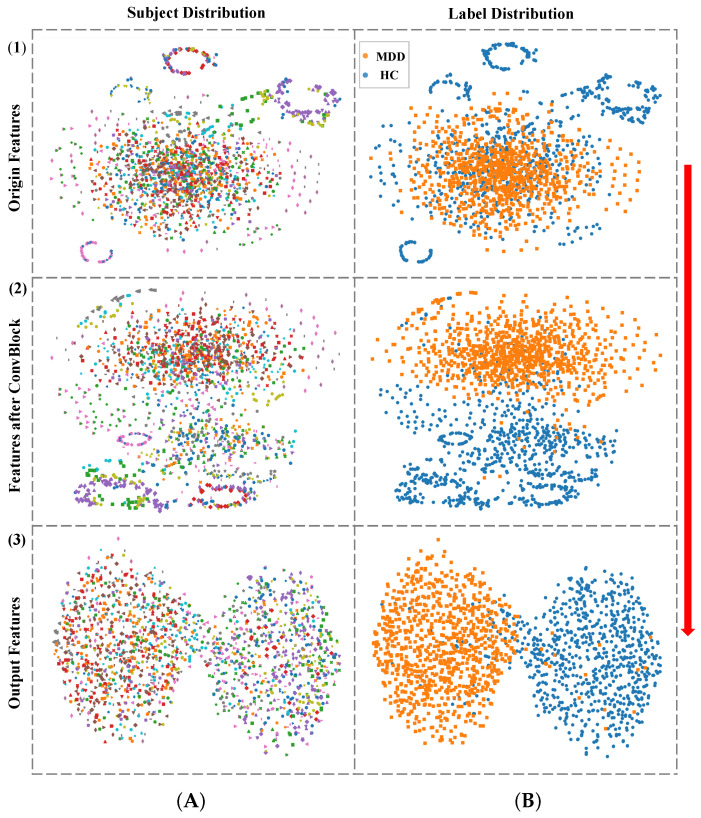
Feature visualization results. From top to bottom: (**1**) original features, (**2**) features after the ConvBlock module, and (**3**) model output features. From left to right: (**A**) subject distribution and (**B**) label distribution. In subject distribution plots, different subjects are represented by unique colors and shapes. In category distribution plots, MDD samples are denoted by orange squares, while HC samples are denoted by blue circles.

To evaluate whether the proposed model can extract subject-independent features and effectively delineate the boundary between MDD and HC, 30 samples were randomly selected from each subject in the original dataset for feature visualization using the T-SNE method [47]. T-SNE was used to visualize the features of the original input data, the features extracted after the ConvBlock module, and the features extracted by the entire model in terms of subject and category (MDD or HC) distribution, as shown in Figure 6. From top to bottom, the rows represent the original features, features after the ConvBlock module, and model output features, respectively. From left to right, the columns represent subject distribution and label distribution. In the subject distribution, different subjects are represented by unique colors and shapes; in the label distribution, MDD and HC are represented by orange squares and blue circles, respectively.

From the subject distribution, the original features of subjects are relatively scattered, with some individual subjects forming isolated ring-like clusters, indicating significant differences among some subjects compared to others. After passing through the ConvBlock module, the overlap of features between subjects increases, but some of the original ring-like clusters still remain. From the lable distribution after the ConvBlock module, it can be observed that the ConvBlock module does not focus on subject-specific differences but instead extracts features that are independent of subjects and beneficial for distinguishing MDD from HC, aligning with the intended design.

From the label distribution, the original features of different categories have significant overlap. After being processed by the model, features of different categories cluster distinctly, forming separable boundaries. This demonstrates that the proposed model successfully extracts subject-independent features for recognizing unseen individuals.

### 4.7. Limitations

Although the results presented in this study are promising, several limitations need to be considered. Firstly, based on the feature outputs from the model in Figure 6, the separation of features between MDD and HC groups is not complete, with a small overlap. This indicates that there are some features that remain ambiguous and contribute to misclassification. This is consistent with the classification performance observed in the actual tests, suggesting that the model’s performance on the Mumtaz2016 dataset can be further optimized. Secondly, although the Mumtaz2016 dataset is widely used in EEG-based depression studies, its relatively small sample size and lack of diversity, combined with the use of only resting-state EEG data and no task based EEG data, may introduce bias.

### 4.8. Future Works

Future directions could explore domain generalization techniques to enhance the model’s ability to adapt to different patient populations and clinical settings. A key to improving model performance is enabling the model to learn universal features across different subjects for automatic patient diagnosis. Due to individual differences in EEG signals, there is a domain shift between the source domain (training set) and the target domain (test set). This issue can be alleviated by domain generalization (DG) techniques, which allow the model to learn universal features across different domains by clustering various individuals or groups to form distinct domains. Furthermore, magnetoencephalography (MEG), as an emerging detection modality, has a spatial resolution advantage over EEG and can provide richer information. Future research could explore the use of MEG signals for depression diagnosis.

To facilitate the clinical application of automatic MDD diagnostic methods, it would be beneficial to integrate these methods into existing clinical pathways to provide objective and rapid assessments that simplify the MDD diagnostic process. However, challenges associated with clinical adoption must be addressed. Firstly, compatibility with traditional MDD diagnostic methods, such as combining clinical assessments and psychological scales, is essential. Secondly, healthcare institutions may need to employ relevant technical staff or rely on external experts. Hospitals will also need to invest in technical equipment and develop specialized personnel. Additionally, EEG-based diagnostics involve the protection of patient EEG data, requiring the assurance of data security and regulatory compliance, which is a critical issue to resolve during implementation. Overcoming these barriers is crucial for the successful integration of this model into routine clinical practice.

## 5. Conclusions

In this study, we proposed a deep learning diagnostic model, TSF-MDD, which integrates temporal, spatial, and frequency-domain information for diagnosing MDD using EEG data. A novel data reconstruction scheme was designed to create a 4D data structure that enables the extraction of fused information across time, space, and frequency domains. The reconstructed data were strictly trained and tested using subject-independent methods. First, we conducted hyperparameter sensitivity experiments to explore the impact of key hyperparameters on model performance, including the number of filters and kernel sizes in 3D-CNN and the dimensions of capsule vectors in the capsule network. Subsequently, ablation experiments were performed to demonstrate the contribution of the capsule network to the model. Next, comparative experiments were carried out in two aspects. On one hand, we compared different input data formats to show that the proposed temporal–spatial–frequency (TSF) data structure outperformed inputs with only temporal or temporal–spatial data. On the other hand, we evaluated the model on the public dataset Mumtaz2016 against other existing models, achieving state-of-the-art performance. Finally, we analyzed the distribution of extracted features using T-SNE visualization, demonstrating that the proposed model effectively captures subject-independent features, making it capable of identifying unseen individuals.

## Figures and Tables

**Table 1:** Various research results under different dataset partitioning strategies.

Dataset Partitioning Strategy	Source	Method	Dataset	Accuracy (%)
Subject-dependent	Mohammed et al. (2023) [14]	FPSE	Private dataset	99.11
	Seal et al. (2021) [15]	DeepNet	Private dataset	99.37
	Dang et al. (2020) [16]	MPWD + MDCNN	Mumtaz2016	97.27
	Saeedi et al. (2021) [17]	1DCNN-LSTM, 2D-CNN-LSTM	Mumtaz2016	99.24
	Sharma et al. (2023) [18]	STFT + CNN + LSTM	Mumtaz2016	99.90
	Lei et al. (2022) [19]	InceptionNet	Private dataset	96.88
	Wang et al. (2021) [20]	Semi-supervised-GCN	MODMA	92.23
	Yang et al. (2023) [21]	GTSAN	MODMA	97.56
Subject-independent	Seal et al. (2021) [15]	DeepNet	Private dataset	91.40
	Li et al. (2020) [8]	functional connectivity + CNN	Private dataset	80.74
	Xia et al. (2023) [23]	self-attention + CNN	Mumtaz2016	91.06
	Chen et al. (2022) [24]	SGP-SL	MODMA	84.91

**Table 2:** Detailed parameters of the proposed model.

Module	Layer	Filter	Activate Function	Optional Parameters	Output Shape
Input	-	-	-	-	(None, 5, 1280, 5, 5)
ConvBlock	Conv3D	32, (512, 3, 3)	-	stride = (16, 1, 1)	(None, 32, 49, 3, 3)
	BatchNormalization3d	-	-	-	(None, 32, 49, 3, 3)
	Activation	-	ReLU	-	(None, 32, 49, 3, 3)
	Concatenate	-	-	-	(None, 392, 3, 3)
	Conv2D	512, (1, 1)	ReLU	-	(None, 512, 3, 3)
	BatchNormalization2d	-	-	-	(None, 512, 3, 3)
	Activation	-	ReLU	-	(None, 512, 3, 3)
PrimaryCaps	Conv2D	32, (3, 3)	-	capsule_dims = 8	(None, 128, 1, 1) × 8
	Reshape	-	-	-	(None, 128, 1) × 8
	Concatenate	-	-	-	(None, 128, 8)
EEGCaps	Dynamic routing	-	Squash	capsule_dims = 16, num_capsules = 2	(None, 2, 16)
	Activation	-	Softmax	-	(None, 2)

**Table 3:** Results of model ablation experiments.

Model	Acc (%)	Pre (%)	Rec (%)	F1 (%)
Conv-FC	91.3	89.8	93.3	91.2
TSF-MDD	**92.1**	**90.0**	**94.9**	**92.4**

**Note:** Bold values indicate the best performance for each metric.

**Table 4:** Comparison of different data input types.

Input Data Type	Method	Acc (%)	Pre (%)	Rec (%)	F1 (%)
Temporal	EEGNet	85.0	83.1	89.2	84.0
Temporal–spatial	TS-MDD	88.3	87.3	89.1	88.0
Temporal–spatial–frequency	TSF-MDD	**92.1**	**90.0**	**94.9**	**92.4**

**Note:** Bold values indicate the best performance for each metric.

**Table 5:** Performance comparison of models on the Mumtaz2016 dataset.

Method	Acc (%)	Pre (%)	Rec (%)	F1 (%)
SVM [5]	80.1	83.0	81.0	82.0
EEGNet [46]	85.0	83.1	89.2	84.0
DeprNet [15]	87.1	87.0	89.0	88.0
1D-CNN-LSTM [17]	86.2	87.2	90.5	87.5
InceptionNet [19]	86.3	85.7	88.0	86.5
Self-attention-CNN [23]	90.5	**90.8**	88.0	89.5
**TSF-MDD**	**92.1**	90.0	**94.9**	**92.4**

**Note:** Bold values indicate the best performance for each metric.

## Data Availability

Publicly available data were used for this work. The dataset can be obtained and referred from “Mumtaz, Wajid (2016). MDD Patients and Healthy Controls EEG Data (New). figshare. Dataset”. https://doi.org/10.6084/m9.figshare.4244171.v2 (accessed on 8 January 2024).
